# Efficacy of Electrical Stimulators for Bone Healing: A Meta-Analysis of Randomized Sham-Controlled Trials

**DOI:** 10.1038/srep31724

**Published:** 2016-08-19

**Authors:** Ilyas S. Aleem, Idris Aleem, Nathan Evaniew, Jason W. Busse, Michael Yaszemski, Arnav Agarwal, Thomas Einhorn, Mohit Bhandari

**Affiliations:** 1 Division of Orthopaedics, Department of Surgery, McMaster University, Hamilton, ON, Canada; 2 Department of Clinical Epidemiology and Biostatistics, McMaster University, Hamilton, ON, Canada; 3 Department of Orthopaedic Surgery, University of Michigan, Ann Arbor, MI, USA; 4 Thalmic Labs, Kitchener, ON, Canada; 5 The Michael G. DeGroote Institute for Pain Research and Care, McMaster University, Hamilton, ON, Canada; 6 Department of Anesthesia, McMaster University, Hamilton, ON, Canada; 7 Department of Orthopaedics, Mayo Clinic, Rochester, MN, USA; 8 University of Toronto Faculty of Medicine, Toronto, ON, Canada; 9 Department of Orthopaedic Surgery, New York University Langone Medical Center, New York, NY, USA

## Abstract

Electrical stimulation is a common adjunct used to promote bone healing; its efficacy, however, remains uncertain. We conducted a meta-analysis of randomized sham-controlled trials to establish the efficacy of electrical stimulation for bone healing. We identified all trials randomizing patients to electrical or sham stimulation for bone healing. Outcomes were pain relief, functional improvement, and radiographic nonunion. Two reviewers assessed eligibility and risk of bias, performed data extraction, and rated the quality of the evidence. Fifteen trials met our inclusion criteria. Moderate quality evidence from 4 trials found that stimulation produced a significant improvement in pain (mean difference (MD) on 100-millimeter visual analogue scale = −7.7 mm; 95% CI −13.92 to −1.43; p = 0.02). Two trials found no difference in functional outcome (MD = −0.88; 95% CI −6.63 to 4.87; p = 0.76). Moderate quality evidence from 15 trials found that stimulation reduced radiographic nonunion rates by 35% (95% CI 19% to 47%; number needed to treat = 7; p < 0.01). Patients treated with electrical stimulation as an adjunct for bone healing have less pain and are at reduced risk for radiographic nonunion; functional outcome data are limited and requires increased focus in future trials.

Bone healing is a complex physiological process and is the end goal in the treatment of patients with fractures, surgical osteotomies and spinal fusion procedures. Failure or delays in bone healing often require further intervention and may result in serious morbidity such as increased pain and functional limitations^1^. Secondary procedures to promote bone healing may be invasive, expensive, and result in significant patient morbidity. The socioeconomic burden associated with bone healing complications such as delayed union or nonunion is substantial and includes direct treatment costs as well as personal and societal costs, such as lost wages, decreased productivity and delays returning to work^2^,^3^,^4^.

Electrical stimulation is a popular adjunctive therapy used to promote bone healing across a range of indications^5^,^6^. Basic science research suggests that electrical stimulation enhances the process of bone healing by stimulating the calcium-calmodulin pathway secondary to the upregulation of bone morphogenetic proteins, transforming growth factor-β and other cytokines^3^,^7^,^8^,^9^,^10^,^11^. Clinical evidence to support the use of electrical stimulators for bone healing has been inconclusive. Prior systematic reviews of electrical stimulation have been limited by narrow scope, poor methodologic quality, and a focus on radiographic healing over patient-important outcomes^12^,^13^,^14^,^15^,^16^,^17^,^18^,^19^. We performed a meta-analysis of randomized sham-controlled trials to determine the effect of electrical stimulation on bone healing, focusing on patient-important outcomes.

## Methods

We report this study according to the Preferred Reporting Items for Systematic Reviews and Meta-Analyses (PRISMA) statement^20^ and the protocol for reviews outlined in the *Cochrane Handbook for Systematic Reviews of Interventions*^21^.

### Identification of Studies

We systematically searched MEDLINE, EMBASE, CINAHL, and the Cochrane Library from inception of the database to March 6, 2016. We used MeSH and EMTREE headings in various combinations and supplemented with free text to increase sensitivity (Appendix 1). Manual searches of the reference lists of included trials were conducted to identify any additional articles. We hand-searched major orthopaedic conference proceedings from March 2013 to March 2016 to identify unpublished studies that were potentially eligible.

### Assessment of eligibility

Two authors independently screened all titles and abstracts and applied eligibility criteria to the methods section of potentially eligible trials using an electronic screening form. All discrepancies were resolved by consensus.

We included all studies fulfilling the following criteria:

1) Adult patients >16 years of any sex undergoing operative or nonoperative treatment for a fresh fracture, nonunion, delayed union, osteotomy, or symptomatic spinal instability requiring fusion.

2) Trials comparing direct current (DC), capacitive coupling (CC), or pulsed electromagnetic field therapy (PEMF).

3) Randomized sham-controlled trials (RCT) only^22^.

No restrictions were made for publication date, language, presence or absence of co-interventions, or length of follow-up. Studies using multiple bones in the same patients as the unit of randomization, rather than patients were excluded due to lack of independence^23^.

### Assessment of risk of bias

Two reviewers independently performed outcome-specific assessment of risk of bias using the Cochrane Collaboration’s tool for risk-of-bias assessment^21^. Attempts were made to contact study authors to resolve any uncertainties when required. When the issues bearing on the risk of bias were identical across outcomes within a study, a single risk of bias assessment was reported^24^.

### Data extraction

Two reviewers independently extracted data using a piloted electronic data extraction form. Extracted data included author names, journal names and publication year, funding source, sample size, mean ages and proportion of each sex in treatment and control groups, descriptions of the interventions in each group, all reported outcomes and follow-up times, and loss to follow-up. We attempted to contact study authors for clarification if important data were unclear or not reported.

Radiographic healing was determined according to the methods implemented in each trial. When multiple criteria for union were described, we recorded the most conservative estimate of union. For each trial we determined whether radiographic assessment was blinded or independently assessed and judged by consensus whether the determination of union was reasonable or not reasonable. We converted radiographic union rates to the number of nonunions by subtracting from the total number of patients in each group. For patients already presenting with a nonunion or delayed union, we recorded the number of patients with persistent or on-going nonunions. In trials that reported union based on CT-scan and plain radiographs, we recorded plain film radiographic healing for consistency.

### Statistical Analyses

We calculated agreement for reviewers’ assessments of study eligibility with the Cohen’s kappa coefficient and agreement for assessments of risk of bias using the intraclass correlation coefficient (ICC). Kappa values ≥0.65 were considered adequate^25^.

Among eligible trials we found substantial diversity in the types of bone lesions targeted for treatment. Although baseline bone healing time differs by size of bone and the site of lesion, the biologic process of healing is consistent across all bone lesions^26^,^27^,^28^,^29^ and the effect of electrical stimulation compared with control on the time to bone healing is therefore likely to be similar. We reasoned that pooling trials exploring the effect of electrical stimulation for different bone lesions would increase the generalizability of our results^30^. We explored the validity of this assumption. We further anticipated that different forms of electrical stimulation may produce different effects, and explored this issue.

We utilized the conservative random-effects model of DerSimonian and Laird to pool effect estimates^21^,^31^. Our primary meta-analysis was an intention-to-treat analysis in which all patients were analysed in the groups to which they were originally randomized. We reported pooled estimates as risk ratios (RR) with 95% confidence intervals (CIs). The Absolute Risk Reduction (ARR) was used to calculate the Number Needed to Treat (NNT) when applicable to aid interpretability^32^,^33^. Continuous outcome instruments measuring the same constructs were summarized using mean differences (MDs) with 95% CIs^21^. If standard deviations were not available, they were estimated from trials with similar outcomes^21^,^34^. We transformed pain scores expressed in different units to the 0 to 100 mm visual analogue scale to facilitate pooling as a weighted mean difference. When there were at least 10 studies in a particular meta-analysis, we examined publication bias by using funnel plots comparing sample size versus treatment effect across the included trials^21^. All tests of significance were two-tailed and *p*-values of <0.05 were considered significant.

### Evaluation of heterogeneity

We quantified heterogeneity using the *Χ*^*2*^test for heterogeneity and the *I*^2^ statistic^21^. *I*^2^ values were interpreted according to the Cochrane Handbook^21^ as: 0–30% might not be important, 30–60% may represent moderate heterogeneity, 50–90% substantial heterogeneity and 75–100% considerable heterogeneity. We prespecified the following two subgroup hypotheses to explain potential heterogeneity^35^.

1. Clinical indication: fresh fractures, delayed union or nonunion, spinal fusion, or surgical osteotomy.
2. Type of stimulation: direct current (DC), capacitive coupling (CC), or pulsed electromagnetic fields (PEMF).

For each subgroup, we performed tests for interaction using a chi-square significance test^36^.

### Sensitivity Analyses

Our main reported analysis is a complete case analysis in which participants with missing data were excluded from both the numerator and denominator. To explore the effects of missing outcome data, we performed sensitivity analyses. For the control group, we assumed the event rate to be the same for patients with missing data and those successfully followed; for the treatment group we assumed plausible ratios of event rates in patients with missing data compared with those who were successfully followed at ratios of: 1.5:1, 2:1, and 2.5:1^37^. As such, we tested the robustness of the results of the primary meta-analysis under relatively extreme assumptions with variable degrees of plausibility^37^,^38^. When only total losses to follow-up were reported and not specific numbers of losses in each arm, we assumed that losses in each arm were equal.

Given potential variability in the methods used to evaluate radiographic union^39^,^40^,^41^ we performed three further sensitivity analyses: (1) including only trials in which an independent assessor was used to determine radiographic union; (2) including only trials in which consensus judgment was reasonable with regards to overall determination of union; (3) including only trials that defined union as >70% of bony continuity, or three of four cortices, as the most conservative estimate of bony union.

### GRADE quality assessment and summary of findings

We utilized the GRADE approach to summarize the quality of the evidence for or against the use of electrical stimulation by each outcome. Data from randomized controlled trials were considered high-quality evidence, but could have been rated down according to risk of bias, imprecision, inconsistency, indirectness, or publication bias^42^.

## Results

### Eligible and Included Studies

**Figure 1:**
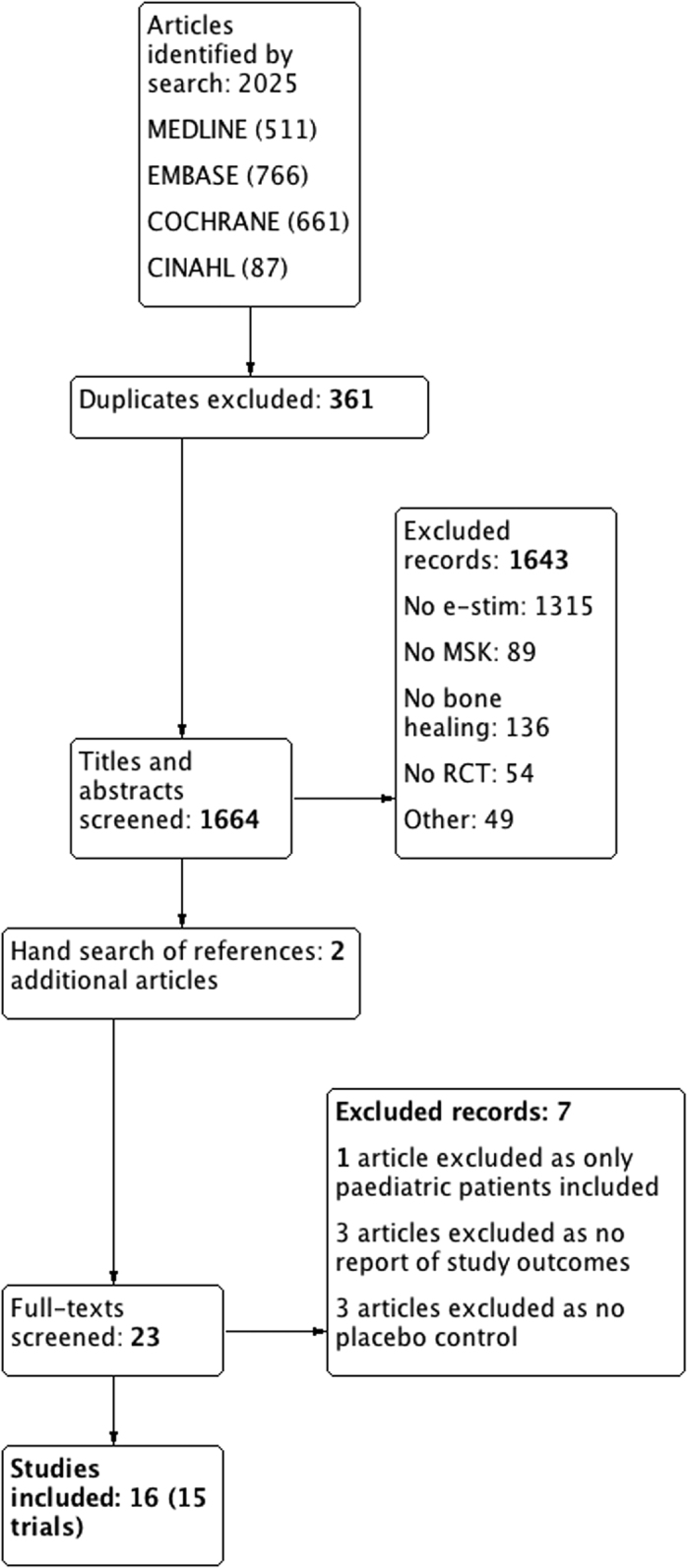
Flow of articles included in the study.

Of 2025 potentially eligible articles, 1664 titles and abstracts were screened and 17 were eligible for our review. However, the authors of one trial^43^ clarified that the same patients were included in a more recent manuscript^44^ and Anderson *et al*. reported different outcomes of the same patient population in two separate publications^45^,^46^. Thus 15 trials that were reported in 16 manuscripts^44^,^45^,^46^,^47^,^48^,^49^,^50^,^51^,^52^,^53^,^54^,^55^,^56^,^57^,^58^,^59^ with a total of 1247 patients were included (Fig. 1). No additional trials from conference proceedings were identified. Agreement between the reviewers for eligibility based on title and abstract screening was very high (kappa = 0.85, 95% CI 0.78–0.93).

### Study characteristics

Mean age of study participants was 45 years in the experimental and control arm. The proportion of male patients in the experimental and control arm was 58.3% and 56.3%, respectively. Mean follow-up was 8.2 (SD 3.4) months for radiographic outcomes and 8.6 (SD 3.7) months for pain and function (Appendix 2).

Four trials included patients undergoing a spinal fusion^45^,^46^,^49^,^52^,^55^, five trials evaluated fresh fracture treatment^44^,^47^,^50^,^51^,^54^,^60^, five trials examined treatment of delayed or nonunions^48^,^56^,^57^,^58^,^59^ and one study included patients undergoing surgical osteotomy^53^. Trials of the appendicular skeleton assessed patients with tibial or femoral fractures^47^,^48^,^53^,^54^,^57^,^59^, femoral neck^44^, scaphoid fractures^50^,^51^, and other long-bone fractures^56^,^58^.

Twelve trials reported use of pulsed electromagnetic field (PEMF) therapy, one trial reported direct current (DC) stimulation and two trials reported continuous current (CC) stimulation. Details with regards to specific stimulator type, frequency and treatment duration for each study are reported in Appendix 3.

Radiographic union was described in all 15 of the included trials (Appendix 4). Consensus judgment with regards to the overall determination of union was deemed to be reasonable in all but four studies^48^,^54^,^56^,^59^. Sharrard^57^ reported results of radiographic union read by both orthopaedic surgeons and radiologists separately.

Pain was reported in four trials using either the Visual Analogue Scale (VAS)^44^, Dallas Pain Questionnaire (DPQ)^46^ or a categorical pain scale^48^,^57^. The lower and upper limits of the categorical pain scale reported in one study^57^ (with demised single author) was assumed to be 0 to 5 based on the reported mean and standard deviations and a statistical simulation. Functional outcome was reported using components of the Short Form 36 (SF-36) health survey in two trials^46^,^47^.

### Risk of bias

**Figure 2:**
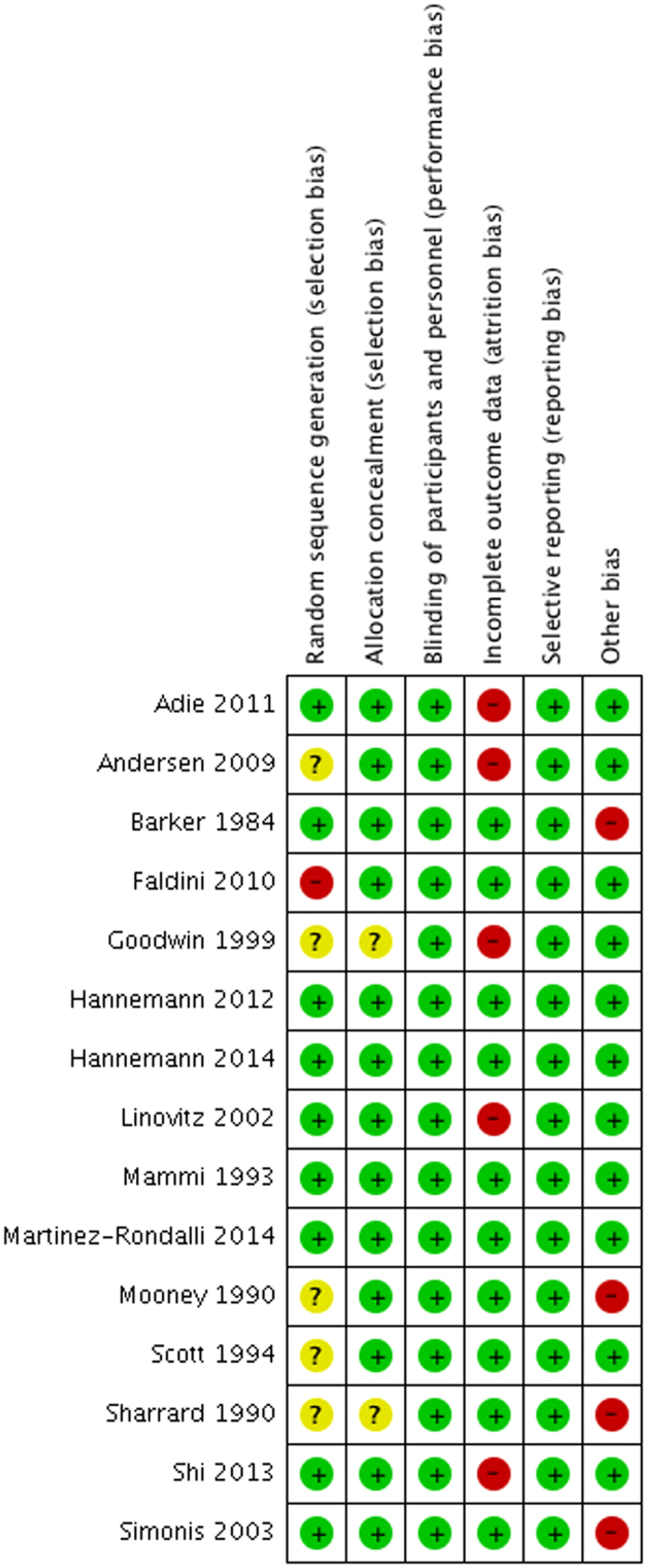
Risk of bias summary: review authors’ judgments about each risk of bias item for included trials. Green circles indicate low risk of bias and red circles indicate high risk of bias.

**Figure 3:**
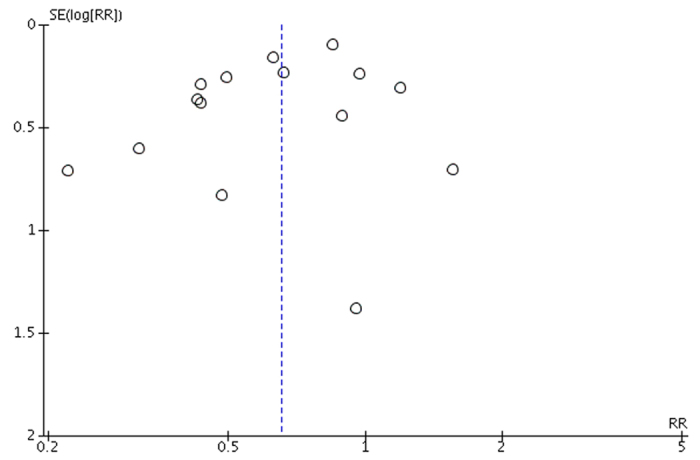
Funnel plot of Standard Error (log(relative risk)) against relative risk to assess for publication bias.

Risk of bias assessments are presented in Fig. 2. The funnel plot for radiographic nonunion at last follow-up was symmetric and did not suggest publication bias (Fig. 3)^21^.

### Pain and function

**Figure 4:**
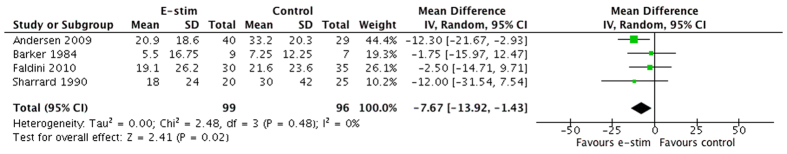
Pooled pain score (mean difference).

Pain was reported across four trials^44^,^46^,^48^,^57^ including a total of 195 patients. The pooled estimate of effect between electrical stimulation and sham control showed a statistically significant difference in pain (MD on the 100 mm visual analogue scale = −7.67 mm, 95% CI −13.92 to −1.43; p = 0.02; I^2^ = 0%; Fig. 4). In the GRADE quality assessment (Table 1) pain was rated as moderate quality due for imprecision given that the 95% CI includes values below and above the minimal important difference (MID) of 10 mm^61^. We found no evidence to support a difference in treatment effect due to treatment indication (interaction p = 0.41) or stimulator type (interaction p = 0.19).

**Figure 5:**
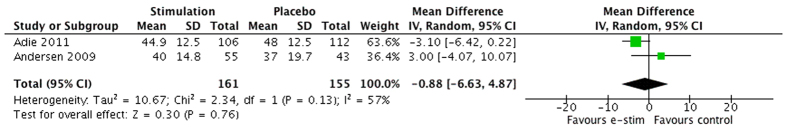
Pooled functional outcome data (mean difference).

Functional outcomes were compared in two trials that reported SF-36 scores (n = 316 patients). The pooled estimate of effect between electrical stimulation and control was not statistically significant (MD −0.88, 95% CI −6.63 to 4.87, p = 0.76) (Fig. 5). In the GRADE quality assessment, functional outcome was rated as low quality evidence due to risk of bias (high losses to follow-up in both studies^46^,^47^) and inconsistency due to unexplained heterogeneity (I^2^ = 57%)^62^.

### Radiographic nonunion

**Figure 6:**
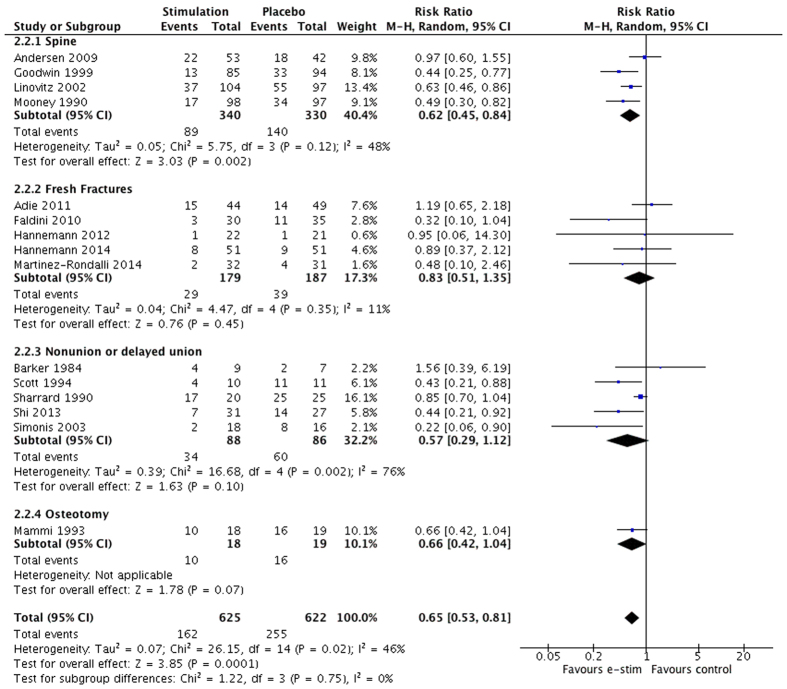
Radiographic nonunion at last reported follow-up to 12 months with subgroups by indication.

Radiographic nonunion was compared across 15 trials with 1247 patients. The pooled estimate of effect between electrical stimulation and sham controls at last reported follow-up up to 12 months found that electrical stimulation reduced the relative risk for nonunion or persistent nonunion by 35% (RR 0.65, 95% CI 0.53 to 0.81, p < 0.01, moderate certainty) and the absolute risk by 15%. Overall between-study heterogeneity was moderate (I^2^ = 46%; p = 0.02) (Fig. 6). Interpreted another way, for every 7 patients treated with electrical stimulation, 1 nonunion or persistent nonunion could be averted (NNT = 7). In the GRADE quality assessment radiographic nonunion was rated as moderate quality evidence due to indirectness (Table 1). We found no evidence to support a difference in treatment effect due to treatment indication (interaction p = 0.75) or stimulator type (interaction p = 0.05) **(Appendices 5 and 6)**. An analysis conducted with the spine studies removed still showed a significant pooled treatment effect in favor of electrical stimulation for acute fracture, nonunion or delayed union, and osteotomy (RR 0.68, 95% CI 0.50 to 0.91, p = 0.01).

### Sensitivity Analyses

Our complete case analysis showed a significant difference (RR 0.66, 95% CI 0.55 to 0.80) that remained robust when assumed nonunion rates in patients with missing data were 1.5:1 and 2:1. When the assumed nonunion rates in patients with missing data went up to 2.5:1 the pooled estimate of effect was no longer significant (Appendix 7).

In only trials in which an independent assessor was used to determine union, a significant difference in favor of electrical stimulation was found (RR 0.69, 95% CI 0.54 to 0.87, p < 0.01). Trials in which consensus judgment deemed the definition and assessment as reasonable showed a significant difference in favor of electrical stimulation (RR 0.68, 95% CI 0.55 to 0.85, p < 0.01). Finally, the most conservative estimate in only those trials^44^,^47^,^50^,^51^,^52^,^53^,^57^,^58^ explicitly defining union as >75% of bony continuity also favored electrical stimulation (RR 0.73, 95% CI 0.58 to 0.91, p < 0.01).

## Discussion

Our systematic review and meta-analysis of eligible randomized controlled trials found moderate quality evidence for electrical stimulation in reducing patient-reported pain and radiographic nonunion or persistent nonunion. Low-quality functional outcome data showed no difference with electrical stimulation compared to sham treatment.

Our findings are strengthened by our comprehensive search and broad clinical eligibility criteria, and by including only randomized sham-controlled trials. We hypothesized the effect of electrical stimulation on bone healing would be similar across different types of stimulation and different clinical lesions, and our subgroup analyses support this assumption. We found no evidence to support a difference in treatment effect due to treatment indication (interaction p = 0.75). In keeping with other orthopaedic trials of bone healing, most trials reported only surrogate end points. Limited reporting of patient-important outcomes is highlighted in this review and has been identified as a significant problem in the surgical literature^63^,^64^. The calculation of Minimally Important Differences (MIDs) can further aid the interpretation of treatment effects, but they are often context- or instrument- specific and may have limited generalizability across clinical indications or varying pain measures^65^,^66^. Although we found the mean difference in pain statistically significant, it is possible that this may not represent a difference important to patients^67^.

A Cochrane review published in 2011 reported non-significant differences for electrical stimulation in improving union rates in four trials involving 125 patients^15^. Two reviews done in 2014 also showed an inconclusive benefit of electrical stimulation. Hannemann *et al*.^51^,^60^ performed a systematic review evaluating the effects of low-intensity pulsed ultrasound (LIPUS) and electrical stimulation specifically in acute fractures; results for LIPUS and electrical stimulation, however, were pooled together and not reported separately. In 2013, Tian *et al*. conducted a meta-analysis looking at the efficacy of various types of electrical stimulation on spinal fusion^18^. Randomized trials and observational trials were, however, combined to provide a pooled estimate and no assessment of methodological quality or risk of bias was performed.

A previous review performed by our group in 2008^16^ specifically assessed long-bone fracture healing and failed to show a significant impact of electrical stimulation on radiographic healing, and inconsistent results for pain relief. In 2014^14^, we performed a systematic review and network meta-analysis to indirectly comparing low-intensity pulsed ultrasonography (LIPUS) and electrical stimulation. Results were pooled separately by 3, 6 and 12-month time points and a borderline significant effect in improving union rates in nonunions or delayed unions at 3 months with electrical stimulation but not at 6 or 12 months was seen. Two trials included in that review, however, were found to have used the same patient groups on contact with the authors^43^,^44^. The present review’s results differ given that our interpretation of the evidence is based upon the addition of 6 recent trials (424 patients) relating to fresh fractures, nonunions/delayed unions and osteotomies^44^,^47^,^50^,^51^,^54^,^58^ (Table 2). Moreover, we restricted eligible trials to only sham-controlled randomized trials and assessed both patient-important and radiographic outcomes. Finally, we broadened our eligibility criteria to include bone healing in spinal fusions and tested the assumption regarding similarity of treatment effect using a formal test of interaction. The addition of this information suggests that electrical stimulation for bone healing may improve rates of radiographic union and produce modest but clinically significant improvements in pain relief.

### Implications for clinical practice and research

A survey of 450 Canadian trauma surgeons in 2008 (response rate 79%) demonstrated that 23% of surgeons used electrical bone stimulators to accelerate bone healing^68^. Our findings support electrical stimulation as an adjunctive modality for radiographic bone healing and reduction in pain. Large trials of high methodological quality focusing on patient important outcomes are needed to establish the effectiveness of electrical stimulation on functional outcomes^69^.

## Conclusions

This systematic review and meta-analysis found that patients treated with electrical stimulation as an adjunct for bone healing have significantly less pain and experience lower rates of radiographic nonunion or persistent nonunion. No difference was seen with regards to functional outcomes in a limited number of trials. Future trials focusing on functional outcomes to identify appropriate indications and ideal patient selection are warranted.

## Additional Information

**How to cite this article**: Aleem, I. S. *et al*. Efficacy of Electrical Stimulators for Bone Healing: A Meta-Analysis of Randomized Sham-Controlled Trials. *Sci. Rep.* **6**, 31724; doi: 10.1038/srep31724 (2016).

## Supplementary Material

Supplementary Information

## Figures and Tables

**Table 1:** Combined GRADE and summary of findings table.

Quality assessment							Number of patients		Effect		Quality
# of trials	Outcome	Risk of bias	Inconsistency	Indirectness	Imprecision	Other considerations	E-stim	Placebo	Relative	Absolute	
									(95% CI)	(95% CI)	
15	Radiographic nonunion	Not serious	Not serious	Serious^4^	Not serious	None	162/625 (25.9%)	255/622 (41.0%)	RR 0.65 (0.53 to 0.81)	143 fewer per 1000 (from 78 fewer to 193 fewer)	⊕⊕⊕⊖ MODERATE
								35.0%		123 fewer per 1000 (from 73 fewer to 163 fewer)	
4	Pain	Not serious	Not serious	Not serious	Serious^3^	Not serious	307	305	—	SMD 0.34 lower (0.62 lower to 0.05 lower)	⊕⊕⊕⊖ MODERATE
2	Functional outcome	Serious^1^	Serious^2^	Not serious	Not serious	None	161	155	—	SMD 0.07 higher (0.33 lower to 0.48 higher)	⊕⊕⊖⊖ LOW

SMD–standard mean difference, RR–relative risk Rated down primarily due to incomplete outcome data (attrition bias) and selective reporting (reporting bias).

^1^Rated down primarily due to incomplete outcome data (attrition bias).

^2^Unexplained heterogeneity, I^2^ = 81%.

^3^95% CI includes values below and above the minimal important difference (MID).

^4^Surrogate outcome.

**Table 2:** Trials included in previous meta-analyses and the present study.

Meta-Analysis Study	1	2	3	4	5	6	7	8	9	10	11	12	13	14	15	16	17	18	19	20	21	22
Akai *et al*.^13^	X	X	X	X	X	X	X	X	X	X	X											
Mollon *et al*.^16^	X			X	X			X				X		X								
Griffin *et al*.^15^	X			X				X						X								
Ebrahim *et al*.^14^	X			X				X						X			X	X	X	X		
Hannemann *et al*.^51^,^60^																	X	X	X			
Aleem *et al*.	X		X	X	X			X			X		X	X	X	X	X	X	X	X	X	X

Barker *et al*.^48^, (2) Kane *et al*. (1988), (3) Mooney *et al*.^55^, (4) Sharrard *et al*.^57^, (5) Mammi *et al*.^53^, (6) Kennedy *et al*. (1993), (7) Traina *et al*.^53^, (8) Scott *et al*.^56^, (9) Capanna *et al*. (1994), (10) Rogozinski *et al*. (1996), (11) Goodwin *et al*.^49^, (12) Betti *et al*.^43^, (13) Linovitz *et al*.^52^, (14) Simonis *et al*.^59^, (15) Andersen *et al*. (Part 1)^46^, (16) Andersen *et al*. (Part 2)^45^, (17) Faldini *et al*.^44^, (18) Adie *et al*.^47^, (19) Hannemann *et al*.^50^, (20) Shi *et al*.^58^, (21) Hannemann *et al*.^51^,^60^, (22) Martinez-Rondanelli^54^.
