# Supplementary material for: Efficacy of Electrical Stimulators for Bone Healing: A Meta-Analysis of Randomized Sham-Controlled Trials
Source: Sci Rep. 2016 Aug 19;6:31724. doi: 10.1038/srep31724 (PMC4990885; doi:10.1038/srep31724)
Supplement: Supplementary Information [file srep31724-s1.pdf]

Efficacy of Electrical Stimulators for Bone Healing:

A Meta-Analysis of Randomized Sham-Controlled Trials

Ilyas S. Aleem*[1,2], Idris Aleem[3], Nathan Evaniew[1,2], Jason W. Busse[2,4,5], Michael Yaszemski[6], Arnav Agarwal[7], Thomas Einhorn[8] and Mohit Bhandari[1,2]

[1]Division of Orthopaedics, Department of Surgery, McMaster University, Hamilton, ON; [2]Department of Clinical Epidemiology and Biostatistics, McMaster University, Hamilton, ON; [3]Thalmic Labs, Kitchener, ON; [4] The Michael G. DeGroote Institute for Pain Research and Care, McMaster University, Hamilton, ON; [5] Department of Anesthesia, McMaster University, Hamilton, ON; [6]Department of Orthopaedics, Mayo Clinic, Rochester, MN; [7]University of Toronto Faculty of Medicine, Toronto, ON; [8]Department of Orthopaedic Surgery, New York University Langone Medical Center, New York, NY

Appendix 1. Search Strategy (conducted March 6, 2016)

MEDLINE	EMBASE	COCHRANE	CINAHL
1. Electric$ stimulation.mp. or exp Electric Stimulation/<br>2. exp Electric Stimulation Therapy/<br>3. Electric stimulator.mp.<br>4. exp Electromagnetic Fields/ or Bone growth stimulation.mp.<br>5. Bone growth stimulator.mp.<br>6. Bone stim$.mp.<br>7. Direct current.mp.<br>8. Capacitive coupling.mp.<br>9. Inductive coupling.mp.<br>10. Magnetic field therapy.mp. or exp Magnetic Field Therapy/ or exp Electromagnetic Fields/<br>11. Pulsed electromagnetic field$.mp.<br>12. Combined magnetic field$.mp.<br>13. Electrical stimulator.mp.<br>14. 1 or 2 or 3 or 4 or 5 or 6 or 7 or 8 or 9 or 10 or 11 or 12 or 13<br>15. Spin$ fusion.mp.<br>16. exp Lumbar Vertebrae/ or exp Spinal Fusion/ or exp Cervical Vertebrae/ or exp Thoracic Vertebrae/<br>17. Spin# surgery.mp.<br>18. exp Spine/ or Spine.mp.<br>19. exp Fractures, Ununited/<br>20. Nonunion.mp.<br>21. Non-union.mp.<br>22. Delayed union.mp.<br>23. exp "Bone and Bones"/<br>24. exp Orthopedic Procedures/ or	1. Electric$ stimulation.mp. or exp electrostimulation/<br>2. functional electrical stimulation/ or Electrical stimulation therapy.mp. or exp electrostimulation therapy/<br>3. Electric$ stimulator.mp.<br>4. Bone growth stimulator.mp. or exp bone growth stimulator/<br>5. exp direct current/<br>6. capacitive coupling.mp.<br>7. inductive coupling.mp.<br>8. Magnetic field therapy.mp. or exp magnetotherapy/<br>9. Electromagnetic fields.mp. or exp electromagnetic field/<br>10. Pulsed electromagnetic field$.mp. or exp pulsed electric field/<br>11. combined magnetic field$.mp.<br>12. 1 or 2 or 3 or 4 or 5 or 6 or 7 or 8 or 9 or 10 or 11<br>13. thoracic spine/ or thoracolumbar spine/ or cervical spine/ or lumbar spine/ or spine fracture/ or exp spine fusion/ or spine fusion implant/ or exp spine/ or exp spine surgery/<br>14. exp fracture healing/ or exp fracture nonunion/ or exp fracture fixation/ or exp fracture treatment/ or exp fracture/<br>15. Nonunion.mp. or exp pseudarthrosis/	#1 MeSH descriptor: [Electric Stimulation] explode all trees<br>#2 Bone growth stimulator$<br>#3 Electromagnetic fields<br>#4 Direct current<br>#5 Capacitive coupling<br>#6 Inductive coupling<br>#7 Magnetic field therapy<br>#8 Spinal fusion<br>#9 Spine surgery<br>#10 Fracture<br>#11 Nonunion<br>#12 Delayed union<br>#13 Fracture healing<br>#14 #1 or #2 or #3 or #4 or #5 or #6 or #7<br>#15 #8 or #9 or #10 or #11 or #12 or #13<br>#16 #14 and #15	S16. S10 AND S14 AND S15<br>S15. S6 OR S7 OR S8 OR S9 OR S11 OR S12 OR S13<br>S14. S1 OR S2 OR S3 OR S4 OR S5<br>S13. (MH "Orthopedic Surgery+")<br>S12. "bone"<br>S11. (MH "Spine+")<br>S10. (MH "Randomized Controlled Trials") OR (MH "Clinical Trials+")<br>S9. (MH "Fractures, Ununited+") OR "nonunion"<br>S8. (MH "Fracture Healing") OR (MH "Spinal Fractures+")<br>S7. "spine surgery"<br>S6. (MH "Spinal Fusion")<br>S5. (MH "Magnet Therapy") OR (MH "Magnetic Fields+") OR "magnetic field therapy"<br>S4. "direct current"<br>S3. "bone stimulator"<br>S2. "bone growth stimulators"<br>S1. (MH "Electric Stimulation+") OR "electrical stimulation or estim"

exp Orthopedics/<br>25. exp Fracture Healing/<br>26. 15 or 16 or 17 or 18 or 19 or 20 or 21 or 22 or 23 or 24 or 25<br>27. randomized controlled trial.pt.<br>28. controlled clinical trial.pt.<br>29. trial.ti.<br>30. placebo.ab.<br>31. Random*.ab.<br>32. 27 or 28 or 29 or 30 or 31<br>33. 14 and 26 and 32<br>34. limit 33 to humans	16. Delayed union.mp. or exp fracture nonunion/<br>17. exp bone/<br>18. exp orthopedics/<br>19. 13 or 14 or 15 or 16 or 17 or 18<br>20. trial.ti.<br>21. placebo.ab.<br>22. random*.ab.<br>23. randomized controlled trial/<br>24. exp controlled clinical trial/<br>25. 20 or 21 or 22 or 23 or 24<br>26. 12 and 19 and 25<br>27. limit 26 to human		

Appendix 2. Baseline characteristics of included trials[1]

Lead Author	Year	Country	Funding	Indication/ Site	Experimental Group				Control Group				Outcomes Reported
					Mean Age (yrs)	% Males	n[2]	Lost/ Missing data	Mean Age (yrs)	% Males	n[3]	Lost/ Missing data	
Adie	2011	Australia	Biomet	Acute fracture/ Tibia	38.5	80	44	85	39.7	84	49	81	Proportion of patients requiring secondary intervention, radiographic union, SF-36 Physical Component Summary, Lower Extremity Functional Scale
Andersen (Part 1)	2009	Denmark	Corporate/ Industry and Federal	Posterolateral fusion/ Lumbar spine	69.3	39.0	53	6	71.4	38.1	42	6	Dallas Pain Questionnaire, SF-36, Low Back Pain Rating Scale pain index, walking distance
Andersen (Part 2)	2009	Denmark	Corporate/ Industry and Federal	Posterolateral fusion/ Lumbar spine	69.3	39.0	55	0	71.4	32.5	43	0	Fusion rate using thin slice CT and radiographs
Barker	1984	England	NR	Nonunion/ Tibia	38	NR	9	0	29.9	NR	7	0	Radiographic union, pain scores
Faldini	2010	Italy	IGEA	Acute fracture/ Femoral neck	69.4	16.6	30	7	68.3	22.8	35	5	Radiographic union, onset of osteonecrosis, VAS pain scale
Goodwin	1999	United States	Biolectron	Fusion/ Lumbar Spine	45	56.5	85	79	40	52.1	94	79	Clinical and radiographic union
Hannemann	2014	Netherlands	Netherlands Organization for Health Research and Development	Acute fracture/ Scaphoid	35	78	51	4	34	75	51	7	Time to clinical and radiographic union, snuffbox tenderness, tenderness with scaphoid compression, wrist range of motion
Hannemann	2012	Netherlands	NR	Acute fracture/ Scaphoid	44.3	75	22	0	37.7	79	21	2	Time to clinical and radiographic union, snuffbox tenderness, tenderness with scaphoid compression, wrist range of motion

[1] NR = Not Reported
[2] Refers to the number analyzed
[3] Refers to the number analyzed

Lead Author	Year	Country	Funding	Indication/ Site	Experimental Group				Control Group				Outcomes Reported
					Mean Age (yrs)	% Males	n[2]	Lost/ Missing data	Mean Age (yrs)	% Males	n[3]	Lost/ Missing data	
Linovitz	2002	United States	Corporate/ Industry	Un-instrumented fusion/ Lumbar spine	56.8	41	104	21	56.6	36.4	97	21	Radiographic union
Mammi	1993	Italy	NR	Osteotomy/ Tibia	62.9	22.2	18	2	61.1	15.8	19	1	Radiographic union
Martinez-Rondanelli	2014	Columbia	Colciencias	Acute Fracture/ Femoral diaphysis	31	82	32	0	29	81	31	1	Radiographic union
Mooney	1990	United States	NR	Interbody fusion (anterior or posterior)/ Lumbar spine	37.9	59.1	98	9	37.6	52.5	97	2	Radiographic union
Scott	1994	England	None	Nonunion/ Femur, tibia	40.6	80	10	1	45.8	73	11	1	Radiographic union
Sharrard	1990	England	None	Delayed union/ Tibia	34.7	70	20	3	45.4	72	25	3	Radiographic union, fracture movement, fracture pain/ tenderness
Shi	2013	China	Third party non-corporate	Delayed union/ Long-bone[4]	41.1	NR	31	3	38.4	NR	27	3	Radiographic union, pain at fracture site
Simonis	2003	United Kingdom	NR	Nonunion/ Tibia	31.7	Very high but NR	18	0	32.3	Very high but NR	16	0	Radiographic union

Appendix 3. Details of intervention and control arms with relative risk of radiographic nonunion in the included trials. PEMF = pulsed electromagnetic fields, DC = direct current, CC = capacitive current

Lead Author	Date	Type of stimulation	Company name	Stimulator frequency (Hz), amplitude, other technical details	Treatment Frequency (hrs / day)	Treatment Duration	Placebo details	Relative Risk of Radiographic Nonunion
Adie	2011	PEMF	Biomet EBI Bone Healing System	NR	Recommended: 10, but at least 6	12 weeks	Identical but Inactive device	1.19 [0.65, 2.18]
Andersen	2009	DC	Biomet Spine SpF-XL IIb Spine Fusion Simulator	40 uA and 100 uA	24	6 months - 1 year after primary operation	Dummy electrodes, identical	0.97 [0.25, 1.17]
Barker	1984	PEMF	Custom design stimulator	15 Hz, circumferential coils fitted around cast, 1.5 mT peak, 5ms burst waveform	10	24 weeks	Dummy device	1.56 [0.39, 6.19]
Faldini	2010	PEMF	Igea BIOSTIM Igeastimulator	75 Hz, 1.3 ms duration, 2mT peak magnetic field	8	90 days	Inactive device	0.17 [0.02, 1.23]
Goodwin	1999	CC	Biolectron Inc SpinalPak (now owned by Biomet)	60 kHz sine wave, 5V peak-peak, 7.1-10.5 mA current. Electrodes placed 10cm apart.	24	Until healed (maximum 9 months)	Inactive device	0.52 [0.24, 1.14]
Hannemann	2014	PEMF	OSSATEC Bone Growth Stimulator	15 Hz, 50 mV pulse amplitude, 5us pulse width, 62 ms burst refractory period	24	6 weeks	Inactive device	0.95 [0.06, 14.3]
Hannemann	2012	PEMF	OSSATEC Bone Growth Stimulator	15 Hz, 50 mV pulse amplitude, 5us pulse width, 62 ms burst refractory period	24	6 or 12 weeks depending on healing	Inactive device	0.89 [0.37, 2.12]
Linovitz	2002	PEMF	Orthologic SpinaLogic	Single coil which produces low-energy combined magnetic fields	0.5 (half hour)	9 months	Inactive device	0.63 [0.46, 0.86]
Mammi	1993	PEMF	Igea BIOSTIM Igeastimulator	75Hz, 3mV, 1.3ms bursts. Two inductively coupled coils	8	60 days	Inactive device	0.66 [0.42, 1.04]

Lead Author	Date	Type of stimulation	Company name	Stimulator frequency (Hz), amplitude, other technical details	Treatment Frequency (hrs / day)	Treatment Duration	Placebo details	Relative Risk of Radiographic Nonunion
Martinez-Rondanelli	2014	PEMF	Custom design stimulator (programmable settings for each patient)	5-105 Hz, 0.5 - 2 mT magnetic field	1	8 weeks	Inactive device	0.7 [0.17, 2.9]
Mooney	1990	PEMF	Custom design stimulator (based on testing on rabbits)	Brace with multiple coils, 1.5 Hz, 1.8 G magnetic field	8	Until healed (although not specifically reported)	Inactive device	0.49 [0.3, 0.82]
Scott	1994	CC	Biolectron OrthoPak Bone Growth Stimulator Systems (now owned by Biomet)	60kHz, 5-10 V, Sine wave 3-6 V. Two stainless-steel disks	NR	Average: 25.4 weeks	Inactive device with very small current flow	0.43 [0.21, 0.88]
Sharrard	1990	PEMF	Custom design stimulator (likely same as Barker 1984)	15 Hz. Two enclosed copper coils, Helmholtz configuration.	12	12 weeks	Inactive device	0.63 [0.41, 0.95]
Shi	2013	PEMF	OSSATEC Orthopulse II	15 Hz, 50 mV pulse amplitude, 5us pulse width, 62 ms burst refractory period (taken from Punt's paper)	8	Until healed (maximum of 3 months)	Inactive device	0.44 [0.21, 0.92]
Simonis	2003	PEMF	Custom design stimulator	23.3 Hz, EMF Force: 150V, Field Intensity: 6A, 1.4 mT	14	26 weeks (6 months)	Inactive device	0.22 [0.06, 0.9]

Appendix 4. Details of bony union definitions, time points assessed, modality of assessment, blinding, use of independent assessor and consensus judgment between reviewers for each included trials. NR = not reported.

Indication	Lead author (Year)	How was union determined/ defined?	Time point (mo)	Modality	Blinding	Independent assessor	Consensus judgment
**Spine**	Andersen (2009)	Continuous bony bridge either between the transverse processes or at the lateral side of the facet joints on at least 1 side or a bilateral fusion of the facet joints	12	Radiographs and CT	Sham device	NR	Reasonable
	Goodwin (1999)	- Fusion success required the presence of mature-appearing, uninterrupted bony masses bilaterally at the fusion levels<br>- ≥ 50% assimilation of graft and vertebrae classified as successful union	12	Radiographs	Sham device	Yes	Reasonable
	Linovitz (2002)	- Solid fusion defined as ≥ 75% bony continuity without motion	9	Radiographs	Sham device	Yes	Reasonable
	Mooney (1990)	- ≥ 50% bony assimilation defined as solid fusion.<br>- In an arthrodesis spanning 2 segments, both levels had to be graded as solidly fused for the patient to be classified as a success.	12	Radiographs	Sham device	Yes	Reasonable
**Fresh Fractures**	Adie (2011)	Union of three of four cortices (75%)	6	Radiographs	Sham device	Yes	Reasonable
	Faldini (2010)	Union was defined as at least 70% of the fracture ends were linked by bone trabeculae.	3	Radiographs	Sham device	NR	Reasonable
	Hannemann (2012)	Union defined as ≥ 75% trabecular bridging	12	CT	Sham device	Yes	Reasonable
	Hannemann	Union defined as ≥ 75% trabecular	12	CT	Sham	Yes	Reasonable

	(2014)	bridging			device		
	Martinez-Rondanelli (2014)	Radiologist classified the fracture healing in one of three alternatives: non-union, partial union, or complete union.	6	Radiographs	Sham device	NR	Not reasonable
**Nonunion/ Delayed union**	Barker (1984)	- Mechanical stressing and limb surface goniometer assessments for mobility in both planes<br>- If lack of mobility suspected, stress radiographs in both planes and tibial behaviour during stressing using image intensification, if lack of mobility was suspected (if both unable to detect movement), fracture defined as clinically united.	6	Radiographs	Sham device	Yes	Not reasonable
	Scott (1994)	- Nonunion defined as at least nine months had elapsed since the injury and there had been no clinical or radiographic signs of progress toward healing, at the site of the fracture, for at least three months before the patient was entered into the study.	9	Radiographs	Sham device	NR	Not reasonable
	Sharrard (1990)	Full union defined as dense and extensive new bone formation across at least 3 of 4 cortices (≥ 75%)	3	Radiographs	Sham device	Yes	Reasonable
	Shi (2013)	Union defined as no pain during joint stressing or during motion at the fracture site, and callus bridging present on 3 of 4 cortices (≥ 75%)	9.6	Radiographs	Sham device	Yes	Reasonable
	Simonis (2003)	- Loss of distinction at the fracture gap,<br>- Cortical bridging<br>- Trabecular bridging.<br>All three criteria required for radiographic union	6	Radiographs	Sham device	No	Not reasonable
**Osteotomy**	Mammi (1993)	- Complete union defined as bridging across entire length of osteotomy site	2	Radiographs	Sham device	Yes	Reasonable

Study or Subgroup	Stimulation Events	Stimulation Total	Placebo Events	Placebo Total	Weight	Risk Ratio M-H, Random, 95% CI
Adie 2011	15	44	14	49	7.6%	1.19 [0.65, 2.18]
Andersen 2009	22	53	18	42	9.8%	0.97 [0.60, 1.55]
Barker 1984	4	9	2	7	2.2%	1.56 [0.39, 6.19]
Faldini 2010	3	30	11	35	2.8%	0.32 [0.10, 1.04]
Goodwin 1999	13	85	33	94	8.1%	0.44 [0.25, 0.77]
Hannemann 2012	1	22	1	21	0.6%	0.95 [0.06, 14.30]
Hannemann 2014	8	51	9	51	4.6%	0.89 [0.37, 2.12]
Linovitz 2002	37	104	55	97	13.4%	0.63 [0.46, 0.86]
Mammi 1993	10	18	16	19	10.1%	0.66 [0.42, 1.04]
Martinez-Rondalli 2014	2	32	4	31	1.6%	0.48 [0.10, 2.46]
Mooney 1990	17	98	34	97	9.1%	0.49 [0.30, 0.82]
Scott 1994	4	10	11	11	6.1%	0.43 [0.21, 0.88]
Sharrard 1990	17	20	25	25	16.1%	0.85 [0.70, 1.04]
Shi 2013	7	31	14	27	5.8%	0.44 [0.21, 0.92]
Simonis 2003	2	18	8	16	2.1%	0.22 [0.06, 0.90]
**Total (95% CI)**		**625**		**622**	**100.0%**	**0.65 [0.53, 0.81]**
Total events	162		255			

Heterogeneity: $Tau^2 = 0.07$; $Chi^2 = 26.15$, df = 14 (P = 0.02); $I^2 = 46\%$
Test for overall effect: Z = 3.85 (P = 0.0001)

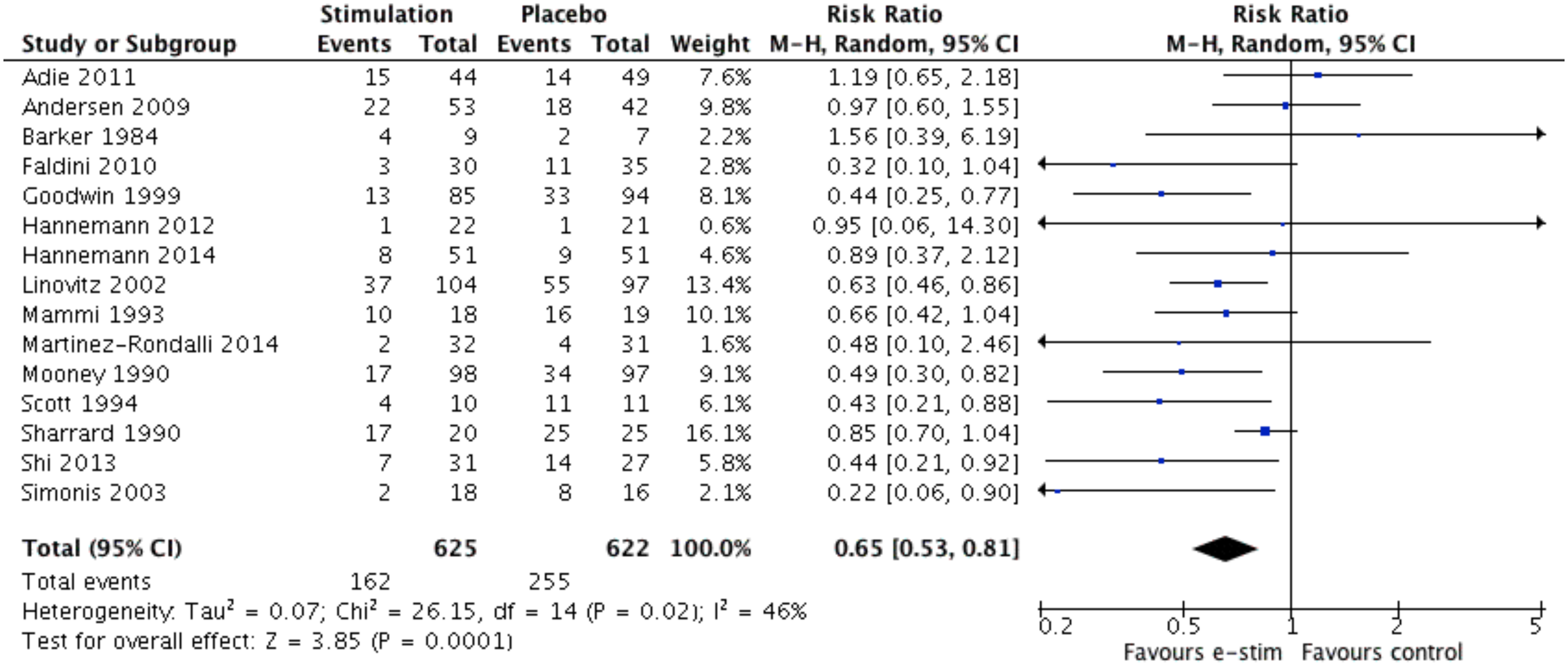

Study or Subgroup	Stimulation Events	Stimulation Total	Placebo Events	Placebo Total	Weight	Risk Ratio M-H, Random, 95% CI
**2.3.1 Pulsed electromagnetic fields (PEMF)**						
Adie 2011	15	44	14	49	7.6%	1.19 [0.65, 2.18]
Barker 1984	4	9	2	7	2.2%	1.56 [0.39, 6.19]
Faldini 2010	3	30	11	35	2.8%	0.32 [0.10, 1.04]
Hannemann 2012	1	22	1	21	0.6%	0.95 [0.06, 14.30]
Hannemann 2014	8	51	9	51	4.6%	0.89 [0.37, 2.12]
Linovitz 2002	37	104	55	97	13.4%	0.63 [0.46, 0.86]
Mammi 1993	10	18	16	19	10.1%	0.66 [0.42, 1.04]
Martinez-Rondalli 2014	2	32	4	31	1.6%	0.48 [0.10, 2.46]
Mooney 1990	17	98	34	97	9.1%	0.49 [0.30, 0.82]
Sharrard 1990	17	20	25	25	16.1%	0.85 [0.70, 1.04]
Shi 2013	7	31	14	27	5.8%	0.44 [0.21, 0.92]
Simonis 2003	2	18	8	16	2.1%	0.22 [0.06, 0.90]
**Subtotal (95% CI)**		**477**		**475**	**76.0%**	**0.67 [0.53, 0.86]**
Total events	123		193			
Heterogeneity: $Tau^2 = 0.06$; $Chi^2 = 19.46$, df = 11 (P = 0.05); $I^2 = 43\%$						
Test for overall effect: Z = 3.20 (P = 0.001)						
**2.3.2 Direct current (DC)**						
Andersen 2009	22	53	18	42	9.8%	0.97 [0.60, 1.55]
**Subtotal (95% CI)**		**53**		**42**	**9.8%**	**0.97 [0.60, 1.55]**
Total events	22		18			
Heterogeneity: Not applicable						
Test for overall effect: Z = 0.13 (P = 0.89)						
**2.3.3 Capacitive coupling (CC)**						
Goodwin 1999	13	85	33	94	8.1%	0.44 [0.25, 0.77]
Scott 1994	4	10	11	11	6.1%	0.43 [0.21, 0.88]
**Subtotal (95% CI)**		**95**		**105**	**14.1%**	**0.43 [0.28, 0.68]**
Total events	17		44			
Heterogeneity: $Tau^2 = 0.00$; $Chi^2 = 0.00$, df = 1 (P = 0.96); $I^2 = 0\%$						
Test for overall effect: Z = 3.68 (P = 0.0002)						
**Total (95% CI)**		**625**		**622**	**100.0%**	**0.65 [0.53, 0.81]**
Total events	162		255			
Heterogeneity: $Tau^2 = 0.07$; $Chi^2 = 26.15$, df = 14 (P = 0.02); $I^2 = 46\%$						
Test for overall effect: Z = 3.85 (P = 0.0001)						
Test for subgroup differences: $Chi^2 = 5.99$, df = 2 (P = 0.05), $I^2 = 66.6\%$						

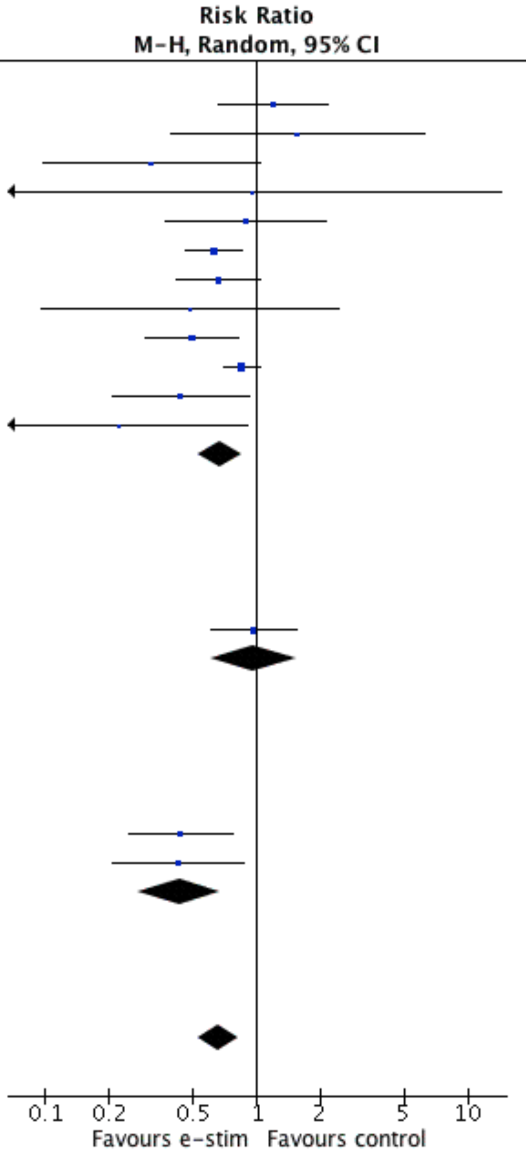

Appendix 7. Sensitivity analysis of missing data at various thresholds for the outcome of radiographic nonunion

Assumed intervention event rates compared to those successfully followed	Pooled Relative Risk (95% CI, *p*-value)
**Complete Case Analysis**	0.65 (0.53 to 0.81, $p < 0.01$)
**1.5:1**	0.71 (0.56 to 0.89, $p < 0.01$)
**2:1**	0.74 (0.58 to 0.96, $p = 0.02$)
**2.5:1**	0.79 (0.60 to 1.03, $p = 0.08$)

PRISMA Checklist

Section/topic	Item No	Checklist item	Reported on page No
**Title**			
Title	1	Identify the report as a systematic review, meta-analysis, or both	1 (Title page)
**Abstract**			
Structured summary	2	Provide a structured summary including, as applicable, background, objectives, data sources, study eligibility criteria, participants, interventions, study appraisal and synthesis methods, results, limitations, conclusions and implications of key findings, systematic review registration number	2<br>Systematic review registration number N/A
**Introduction**			
Rationale	3	Describe the rationale for the review in the context of what is already known	3
Objectives	4	Provide an explicit statement of questions being addressed with reference to participants, interventions, comparisons, outcomes, and study design (PICOS)	3
**Methods**			
Protocol and registration	5	Indicate if a review protocol exists, if and where it can be accessed (such as web address), and, if available, provide registration information including registration number	Review protocol N/A
Eligibility criteria	6	Specify study characteristics (such as PICOS, length of follow-up) and report characteristics (such as years considered, language, publication status) used as criteria for eligibility, giving rationale	4-5
Information sources	7	Describe all information sources (such as databases with dates of coverage, contact with study authors to identify additional studies) in the search and date last searched	4
Search	8	Present full electronic search strategy for at least one database, including	Appendix 1

Section/topic	Item No	Checklist item	Reported on page No
		any limits used, such that it could be repeated	
Study selection	9	State the process for selecting studies (that is, screening, eligibility, included in systematic review, and, if applicable, included in the meta-analysis)	4-5
Data collection process	10	Describe method of data extraction from reports (such as piloted forms, independently, in duplicate) and any processes for obtaining and confirming data from investigators	5-6
Data items	11	List and define all variables for which data were sought (such as PICOS, funding sources) and any assumptions and simplifications made	5-6
Risk of bias in individual studies	12	Describe methods used for assessing risk of bias of individual studies (including specification of whether this was done at the study or outcome level), and how this information is to be used in any data synthesis	5
Summary measures	13	State the principal summary measures (such as risk ratio, difference in means).	6
Synthesis of results	14	Describe the methods of handling data and combining results of studies, if done, including measures of consistency (such as I^2statistic) for each meta-analysis	7-8
Risk of bias across studies	15	Specify any assessment of risk of bias that may affect the cumulative evidence (such as publication bias, selective reporting within studies)	7 (publication bias); 9 (GRADE assessment)
Additional analyses	16	Describe methods of additional analyses (such as sensitivity or subgroup analyses, meta-regression), if done, indicating which were pre-specified	Sensitivity analyses - 8
**Results**			
Study selection	17	Give numbers of studies screened, assessed for eligibility, and included in the review, with reasons for exclusions at each stage, ideally with a flow diagram	9, Figure 1 (page 28)
Study characteristics	18	For each study, present characteristics for which data were extracted (such as study size, PICOS, follow-up period) and provide the citations	10-11, Appendix 2-4
Risk of bias within	19	Present data on risk of bias of each study and, if available, any outcome-	11

Section/topic	Item No	Checklist item	Reported on page No
studies		level assessment (see item 12).	Figure 2-3
Results of individual studies	20	For all outcomes considered (benefits or harms), present for each study (a) simple summary data for each intervention group and (b) effect estimates and confidence intervals, ideally with a forest plot	11-12<br>Figures 4-6<br>Appendices 5-6
Synthesis of results	21	Present results of each meta-analysis done, including confidence intervals and measures of consistency	11-12<br>Figures 4-6<br>Appendices 5-6
Risk of bias across studies	22	Present results of any assessment of risk of bias across studies (see item 15)	11<br>Figures 2-3
Additional analysis	23	Give results of additional analyses, if done (such as sensitivity or subgroup analyses, meta-regression) (see item 16)	12-13<br>Appendix 7
**Discussion**			
Summary of evidence	24	Summarise the main findings including the strength of evidence for each main outcome; consider their relevance to key groups (such as health care providers, users, and policy makers)	Page 14-16
Limitations	25	Discuss limitations at study and outcome level (such as risk of bias), and at review level (such as incomplete retrieval of identified research, reporting bias)	14
Conclusions	26	Provide a general interpretation of the results in the context of other evidence, and implications for future research	17
**Funding**			
Funding	27	Describe sources of funding for the systematic review and other support (such as supply of data) and role of funders for the systematic review	25
